# Meckel’s Extraordinary Complication

**DOI:** 10.4274/balkanmedj.galenos.2020.2019.11.23

**Published:** 2020-04-10

**Authors:** Osman Uzunlu, Yeliz Arman Karakaya

**Affiliations:** 1 Department of Pediatric Surgery, Pamukkale University School of Medicine, Denizli, Turkey; 2 Department of Pathology, Pamukkale University School of Medicine, Denizli, Turkey

A 3-year-old previously healthy girl was admitted to the emergency department because of vomiting, colicky abdominal pain, and abdominal discomfort and distention. When examined physically, she seemed lethargic, dehydrated, and had tachycardia. Her abdominal examination revealed distention, hypoactive bowel sounds, and tenderness in the periumbilical area. Her rectal examination was unremarkable, laboratory findings were normal, and C-reactive protein level was normal. Plain abdominal radiography showed air-fluid levels and dilated loops of the small bowel. Ultrasonography (US) showed distended small bowel loops, and a 5 cm × 3 cm hyperechogenic solid lesion in the subumbilical region. Similar findings were seen using a computed tomography (CT) scan. Neither the US nor the CT scans showed intussusception.

Her previous medical records were unremarkable with no history of surgery to explain the intestinal obstruction.

After treatment with intravenous fluid resuscitation, the patient underwent an urgent operation. A complete intestinal obstruction was observed and was caused by a 10 cm intestinal segment with a substantial intramural hematoma, at approximately 50 cm from the ileocaecal valve (Figure 1). The observed intramural hematoma resulted from hemorrhage of the adjacent Meckel’s diverticulum. The affected intestinal loops with Meckel’s diverticulum were resected, and an end-to-end anastomosis was performed. The postoperative period was uneventful. Histopathological tests revealed that the Meckel’s diverticulum contained ectopic gastric tissue and an extensive intramural hematoma secondary to bleeding from the Meckel’s diverticulum. Upon pathological examination, the etiology of the complete intestinal obstruction was found to be this intramural hematoma. The patient’s family agreed with written informed consent paper.

Meckel’s diverticulum is a true diverticulum that is a remnant of the vitelline duct. It is observed in 2% of the population and is the most common anomaly of the gastrointestinal tract (1). Meckel’s diverticulum is a clinically silent anomaly; however, the related complications may develop in 4%–16% of cases (2). Although gastrointestinal bleeding and diverticulitis are the most frequent clinical complications of Meckel’s diverticulum in childhood, volvulus, intestinal obstruction, and intussusception may also be rarely observed. Intestinal obstruction due to Meckel’s diverticulum may develop because of intussusception, volvulus, diverticulitis, or internal hernias.

Pediatric surgeons generally predict that if a child has an intestinal obstruction, he or she might also display a Meckel’s diverticulum. Meckel’s diverticulum may cause intestinal obstruction for unexpected reasons, such as inverted diverticulum and torsion of the diverticulum etc.

Meckel’s diverticulum accounts for approximately 50% of all lower gastrointestinal bleeding in children. Hemorrhage typically occurs intraluminally; however, atypical hemorrhagic features have been documented, such as free intraperitoneal bleeding, which is quite rare (3). As mentioned above, to the best of the authors’ knowledge, only one other case presenting complications of the Meckel’s diverticulum, with a similar bleeding pattern into the layers of the intestinal wall, was reported in 1981 (4). According to the authors’ knowledge, Meckel’s hemorrhage typically occurs intraluminally. However, in the present case, hemorrhage occurred between the layers of the intestinal wall and caused the hematoma structure to expand into the intraluminal space, which is not a well-known feature of Meckel’s diverticulum.

## Figures and Tables

**Figure 1:**
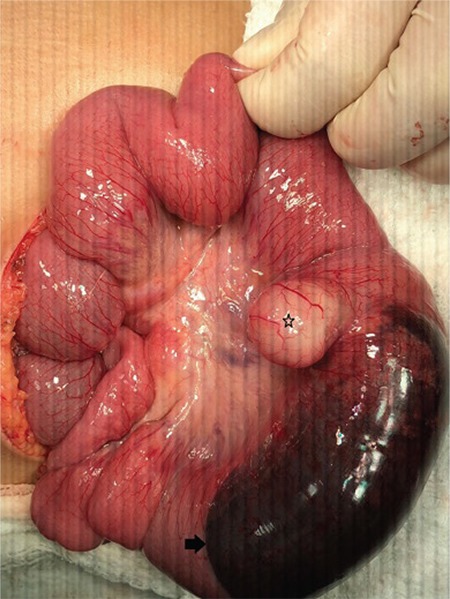
The ileum which contains Meckel's diverticulum with intramural hematoma structure shown here. Star: Meckel's diverticulum, arrow: intramural hematoma adjacent to the diverticulum.
